# Exploring person-centredness in technology-based gait rehabilitation after stroke: A scoping review framework analysis

**DOI:** 10.1177/20552076261443750

**Published:** 2026-04-30

**Authors:** Júlio Belo Fernandes, Diana Vareta, Sónia Fernandes, Ana Chalaça, Ana Silva Almeida, Ana Catarina Maia, Faiza Magsi, Steven Hall, Brendan McCormack

**Affiliations:** 1 Egas Moniz Center of Interdisciplinary Research (CiiEM), 112055 Egas Moniz School of Health & Science, Almada, Portugal; 2 Nurs* Lab, Almada, Portugal; 3 Arrábida Local Health Unit, Hospital de São Bernardo, Setúbal, Portugal; 4 Health Sciences Research Unit: Nursing (UICISA: E), 451281 Nursing School of Coimbra (ESEnfC), Coimbra, Portugal; 5 14722 University of Nevada Las Vegas, Las Vegas, NV, USA; 6 Faculty of Nursing, 3158 University of Alberta, Edmonton, AB, Canada; 7 Susan Wakil School of Nursing and Midwifery Ringgold Standard Institution, 95627 The University of Sydney, Sydney, NSW, Australia; 8 Sydney Policy Lab Ringgold Standard Institution, 95627 The University of Sydney, Sydney, NSW, Australia; 9 Lararutbildning Ringgold Standard Institution, Kristianstad University, Kristianstad, Sweden; 10 Ringgold Standard Institution, Ulster University, Coleraine, UK; 11 Roskilde Ringgold Standard Institution, Zealand-Sjallands Erhvervsakademi-Campus, Roskilde, Denmark

**Keywords:** person-centred practice, person-centred care, stroke, gait rehabilitation, technology-based interventions

## Abstract

**Background:**

Technology-based gait rehabilitation after stroke is expanding, however the extent to which interventions embody person-centred care remains unclear.

**Aims:**

The objective of this review is to explore the extent to which technology-based interventions for gait rehabilitation in stroke survivors reflect the principles of person-centred practice.

**Methods:**

A scoping review was conducted following the Arksey and O’Malley methodological framework and the Preferred Reporting Items for Systematic Reviews and Meta-Analyses extension for Scoping Reviews (PRISMA-ScR) guidelines. A comprehensive search was performed in MEDLINE Complete, CINAHL Complete, Nursing & Allied Health Collection: Comprehensive, and the Cochrane Central Register of Controlled Trials, with the last search update on September 1, 2025. Data charting was aligned with the domains of the PCPF, enabling the identification of person-centredness indicators both explicitly stated in the texts and inferred from their context.

**Results:**

The search identified 1,460 records; after screening, 21 randomised controlled trials were included. Extracted data were mapped to PCPF domains to identify explicit and implicit indicators of person-centredness. Representation was variable: Prerequisites, Practice Environment, Person-Centred Processes, and Outcomes appeared across studies, while Macro Context was absent. Alignment was evident via practitioner expertise and responsive parameter adjustments (Prerequisites), specialised, well-resourced settings (Practice Environment), and personalised goal setting, real-time feedback, and preference-sensitive features (Processes). Regarding outcomes, all studies reported performance-based measures (motor performance); one included biomarker outcomes and two used Patient-Reported Outcome Measures (quality of life). No Patient-Reported Experience Measures were reported.

**Conclusion:**

Technology-focused gait trials remain predominantly biomedical in framing and reporting. Future studies should embed and report explicit person-centred processes and broaden outcomes to include patient-reported experience measures.

## 1. Introduction

Gait impairments are among the most prevalent and debilitating consequences experienced by stroke survivors, often resulting in reduced mobility, impaired functional independence, and lower quality of life.^1,2^ Beyond the biomechanical disruption of walking patterns, gait dysfunction after stroke often embodies a complex interaction between motor deficits, cognitive challenges, emotional responses, motivation, and contextual constraints.^3,4^ Consequently, gait rehabilitation cannot be understood solely as a technical process aimed at restoring movement patterns, but a deeply person-dependent process shaped by individual goals, values, expectations, and lived experiences of recovery.^5^

Rehabilitation strategies targeting gait restoration have evolved significantly over the past decades, with a notable increase in the integration of technology-based interventions into clinical practice.^6^ These interventions, ranging from robotic-assisted gait training and virtual reality environments to wearable devices and functional electrical stimulation, aim to provide personalised, intensive, and engaging approaches to motor recovery.^7–9^

Collectively, these reviews have contributed valuable insights into technological innovation and its potential to support gait recovery. However, despite the growing sophistication of these interventions, existing reviews have largely focused on technological characteristics and clinical outcomes, paying limited attention to how such interventions are conceptualised, delivered, and experienced from the perspective of the person undergoing rehabilitation. Specifically, little is known about the extent to which technology-based gait interventions align with contemporary paradigms of person-centred practice (PCP). This represents a significant gap in the literature, as rehabilitation is not merely concerned with functional gains, but with how individuals make sense of, engage with, and sustain therapeutic processes over time, positioning recovery as a relational, contextual, and meaning-oriented endeavour.^5,10,11^

Person-centredness has emerged as a cornerstone of high-quality healthcare and is particularly relevant in rehabilitation contexts, where interventions often require sustained engagement, behavioural adaptation, and active participation over time.^12,13^ Person-centred rehabilitation emphasises the recognition of the individual as an active partner in care, valuing personal goals, preferences, lived experiences, and social contexts alongside clinical and functional indicators.^14–16^ In the context of gait rehabilitation after stroke, this approach can be especially pertinent, as walking recovery is closely linked to personally meaningful activities such as returning to community participation, resuming valued social and family roles, and regaining autonomy in daily life.^17,18^

Moreover, adherence to training programmes and sustained engagement with rehabilitation are influenced by motivational, emotional, and relational factors.^5,19^ Interventions that fail to acknowledge individual goals, capabilities, and preferences risk being perceived as burdensome, impersonal, or misaligned with what matters most to the person, potentially limiting their effectiveness despite technological sophistication.^5,20^ In contrast, when interventions are embedded within a person-centred philosophy, they may serve as enablers of agency, motivation, and shared ownership of the rehabilitation process.^13,21^ This underscores the need to examine not only technological efficacy, but also the extent to which such interventions support person-centred processes in practice.

It is within this context that the integration of a robust person-centred theoretical framework becomes essential to guide critical analysis and interpretation. To address this dimension, we adopted the Person-Centred Practice Framework (PCPF) of McCance and McCormack,^22^ a model that provides a theoretical and practical foundation for understanding, implementing, and evaluating person-centred care, emphasising that its achievement depends on the alignment of individual, organisational, and relational factors. The framework comprises five interrelated domains: (a) *Macro Context*, referring to the broader sociopolitical, cultural, and economic forces that shape healthcare environments; (b) *Prerequisites*, which denote the professional attributes of healthcare providers; (c) *The Practice Environment*, encompassing the organisational systems, culture, and infrastructure that enable or constrain PCC; (d) *Person-Centred Processes*, including practices such as shared decision-making, authentic engagement, and working with individuals’ beliefs and values; and (e) *Outcomes*, conceptualised as a ‘healthful culture’, reflecting a state in which well-being and quality of care are fostered across individuals, health professionals and organisations. In this regard, outcomes can be understood through complementary but distinct patient-reported perspectives. Patient-Reported Outcome Measures (PROMs) capture individuals’ perceptions of health status, symptoms, functional abilities, and quality of life, reflecting the perceived impact of interventions on outcomes that matter to the person. Patient-Reported Experience Measures (PREMs), in turn, focus on how care is experienced, including communication, therapeutic relationships, involvement in decision-making, and the extent to which care processes are perceived as respectful, supportive, and person-centred. Together, PROMs and PREMs offer a more comprehensive view: the former speaks to *what* changes, while the latter clarifies *how* care is delivered and experienced.^23^

In this review, we critically analyse the evidence through the lens of the PCPF^22^ to evaluate whether, and to what extent, person-centred principles are integrated into technology-based interventions for gait rehabilitation after stroke. Particular attention is given to how the framework’s domains are operationalised (or overlooked) within these interventions.

This theory-based analysis is both timely and necessary. As healthcare systems increasingly adopt technology-driven approaches, there is a risk that the humanistic and relational aspects of care may be overlooked in favour of efficiency and standardisation.^24–26^ Without intentional reflection, technological innovation may inadvertently reinforce task-oriented or protocol-driven practices that marginalise individual experience and agency. Ensuring that innovation does not come at the expense of personhood requires intentional reflection and evaluation using robust conceptual models.

By applying the PCPF to this body of evidence, we aim to generate insights that can inform the design of more comprehensive and adaptable rehabilitation programs. Such programmes can leverage technological advancements while upholding a strong commitment to PCC principles, thereby broadening the definition of what constitutes a “successful” intervention in stroke recovery.

The objective of this review is to explore the extent to which technology-based interventions for gait rehabilitation in stroke survivors reflect the principles of person-centred practice. Specifically, we aim to:

1. Identify how the domains of the PCPF are represented or operationalised in the design, delivery, and evaluation of these interventions.
2. Assess the degree to which person-centred principles are integrated into the technological components and therapeutic strategies used in gait rehabilitation.
3. Highlight gaps, limitations, or opportunities for enhancing the person-centredness of technology-based rehabilitation programs for stroke survivors.

Through this analysis, we hope to contribute to the advancement of rehabilitation science by promoting a more integrated approach that values both technological innovation and the centrality of the person in care delivery.

## 2. Methods

This scoping review followed Arksey and O’Malley’s^27^ five-stage framework. Reporting was guided by PRISMA-ScR^28^ to maximise transparency and completeness. The review was not registered. Data charting was structured according to the domains of the Person-Centred Practice Framework (PCPF),^22^ enabling the systematic identification of reported indicators of person-centredness within the included studies.

### 2.1. Identifying the research question

The review was guided by the research question: *“To what extent do technology-based gait rehabilitation interventions for stroke survivors reflect the principles of person-centred practice as outlined in the PCPF?”* The PCC mnemonic (Population, Concept, Context) was applied in formulating this question to ensure alignment with the study objectives and to provide a clear framework for defining the inclusion and exclusion criteria.^29^

### 2.2. Identifying relevant studies

A search strategy was run using the following databases MEDLINE Complete, CINAHL Complete, Nursing & Allied Health Collection: Comprehensive, and the Cochrane Central Register of Controlled Trials, with the last search update on September 1, 2025.

To maximise sensitivity, the search strategy incorporated Medical Subject Headings (MeSH) along with alternative keywords, structured according to the PCC framework (Table 1). Rather than enumerating specific technologies, which vary widely and evolve rapidly, the strategy intentionally focused on broader gait-related intervention terms. This approach was chosen to capture the full spectrum of technology-based gait rehabilitation interventions, including emerging and hybrid technologies, while reducing the risk of prematurely excluding relevant studies. Importantly, technological diversity was operationalised primarily during the study selection phase rather than at the search stage. All forms of technology-based gait rehabilitation were eligible at screening, provided they met the predefined criteria (Section 2.3). For this review, a technology-based gait rehabilitation intervention was defined as any intervention that relies on digital, mechanical, or electronic systems (including device-based, sensor-driven, robotic, electrical stimulation, virtual/augmented feedback, or neuromodulation approaches) and is explicitly applied to improve gait outcomes after stroke. This two-step approach, broad conceptual searching followed by detailed eligibility screening, enhanced transparency while ensuring comprehensive coverage of the field.

**Table 1.** Search terms and synonyms.

MeSH descriptors	Synomyms
Stroke	Stroke, Cerebrovascular accident, CVA
Gait	Gait, Gait training, Gait rehabilitation
***-***	Interventions, Strategies, Best Practices

The search strategy:

The search strategy was structured into three concept blocks: S1, S2, and S3. The same core search structure was applied consistently across all databases.

The search strategy was defined as follows:

S1: (Stroke OR Cerebrovascular accident OR CVA).

S2: (Gait OR Gait training OR Gait Rehabilitation).

S3: (Interventions OR Strategies OR Best practices).

Combined search: S1 AND S2 AND S3.

### 2.3. Study selection

All retrieved records were exported to Rayyan, an AI-assisted tool for systematic reviews, to support the screening process. Duplicates were removed, and two reviewers independently screened titles and abstracts according to the predefined eligibility criteria (Table 2). Any disagreements were resolved through discussion until a consensus was reached; when consensus could not be achieved, a third reviewer was consulted to support the final decision.

**Table 2.** Eligibility criteria.

​	Inclusion criteria	Exclusion criteria
Population	Stroke survivors aged 18 years or older.	Participants with other health conditions besides stroke or aged under 18 years.
Concept	Technological interventions for gait rehabilitation, regardless of the type of technology (digital, mechanical, or electronic systems), provided they aimed to improve gait.	Studies not addressing gait rehabilitation.
Context	Conducted in a rehabilitation setting.	Conducted outside rehabilitation settings.
Type of evidence source	Randomised controlled trials.	Designs other than randomised controlled trials.

Due to resources and language fluency of the research team, we only included full text articles published in English or Portuguese publish between 2014 and 2025. This time window was chosen to capture research that reflects the most up-to-date and emerging trends in technology-based gait rehabilitation for stroke survivors.

We restricted eligibility to randomised controlled trials because they are the principal design used to validate emerging rehabilitation technologies clinically, typically providing the pivotal evidence base that precedes wider adoption. Moreover, randomised controlled trials offer granular details of interventions, enabling precise extraction of intervention components.

**Figure 1.**
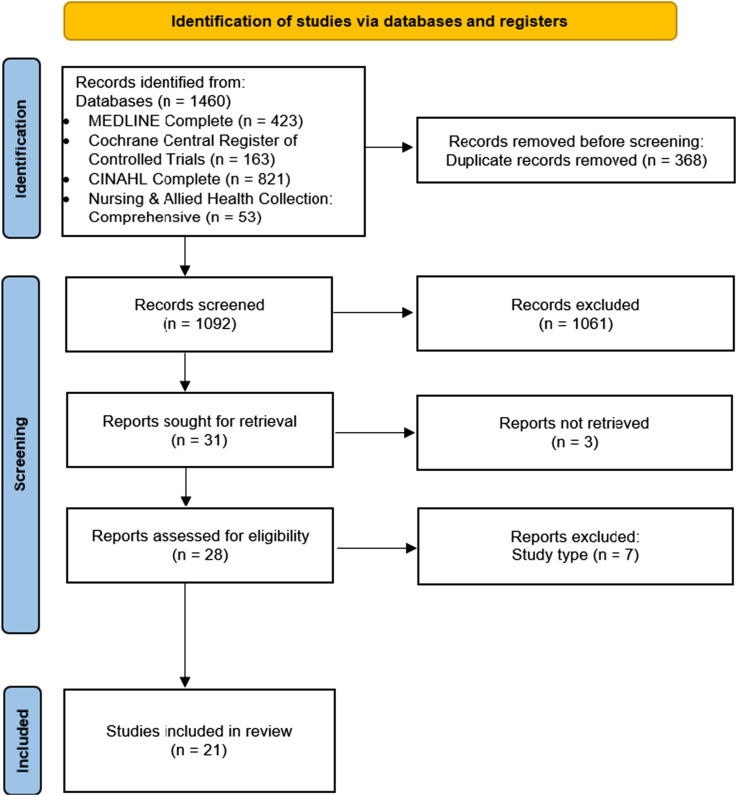
PRISMA flowchart.

The selection process, from the initial search to the final inclusion of studies, is summarised in the PRISMA-ScR flow diagram (Figure 1).

### 2.4. Charting the data

Data from the included studies were systematically extracted using a structured charting form developed for this review. The extraction process captured key bibliographic and methodological details, including:

• Author(s) and year of publication
• Country of origin
• Study population characteristics
• Description of the intervention (type of technology, components, delivery format, and setting)
• Outcomes assessed (both primary and secondary) and main findings
• Any elements identified as aligning with one or more domains of the PCPF.

The PCPF mapping was performed independently by two reviewers based on the information reported in the trial descriptions. In cases where consensus was not reached, a third reviewer was consulted to support resolution.

### 2.5. Collating, summarising, and reporting the results

A thematic analysis was conducted following Braun and Clarke’s^30^ six-phase approach (familiarisation, initial coding, theme development, review, definition, and reporting) using the PCPF as the interpretive map. Text segments were coded for explicit and implicit indicators of person-centredness and mapped to PCPF domains and constructs. Coding was performed independently by two reviewers, discrepancies were resolved by consensus, and an audit trail was maintained to ensure transparency.

## 3. Results

From an initial yield of 1460 records, duplicate removal and title/abstract screening resulted in the exclusion of 1060. Of the studies selected for full-text review, three were not included due to the unavailability of the complete article. This left 28 full texts for detailed assessment, of which 21 satisfied the eligibility criteria and were included in the final synthesis. The remaining studies were excluded at the full-text stage because they did not meet the predefined study design criteria. The complete selection process is presented in Figure 1.

Of these 21 randomised controlled trials, the technologies evaluated spanned treadmill-based paradigms with visual feedback and rhythmic auditory cueing, rhythmic auditory stimulation delivered independently, and treadmills providing real-time visualisation of foot placement; some protocols coupled treadmill training with functional electrical stimulation triggered by tilt sensors. A large subset evaluated robot-assisted gait training, including variants that integrated functional electrical stimulation; additional over-ground systems provided active body-weight support, and ambulatory aids delivered weight-support feedback. Other device-centred approaches comprised whole-body vibration platforms, three-dimensional spine stabilisation systems, and peripheral electrical stimulation delivered through multi-pad or movement-synchronised configurations. Finally, neuromodulation and hybrid modalities were tested, including transcranial direct current stimulation, repetitive transcranial magnetic stimulation combined with visual-feedback cycling, and repetitive transcranial magnetic stimulation paired with augmented-reality, gait-adaptive training.

This technological heterogeneity provides the context for the framework analysis. When mapped to the PCPF, technology-based gait rehabilitation for stroke survivors showed variable but discernible alignment. Four of the framework’s domains *Prerequisites*, *Practice Environment*, *Person-Centred Processes*, and *Outcomes*, were represented to varying extents, while the *Macro Context* domain was absent in all extracted descriptions. The extracted data pertaining to PCPF domains are described herein.

### 3.1. Macro context

Not reported in the extracted descriptions.

### 3.2. Prerequisites

Several interventions demonstrated alignment with the *Prerequisites* domain by requiring professional attributes such as advanced technical competence, capacity for individualised clinical reasoning, and the ability to adapt interventions responsively.

The professionally competent construct is evidenced with clinicians continuously calibrating technological parameters to the individual: guidance forces and body-weight support in robotic devices,^31–39^ real-time tuning in electrical stimulation and rhythmic auditory stimulation,^40–46^ and progressive treadmill adjustments to fatigue and functional capacity.^34,47–49^ These examples illustrate how the interventions relied on skilled practitioners capable of continuous monitoring and adjustment to ensure both safety and optimisation of therapeutic benefit. The constructs of development of interpersonal skills, commitment to the job, clarity of beliefs and values, and knowing self, are not explicitly reported in the included papers.

### 3.3. The practice environment

*The Practice Environment* domain was reflected in settings where organisational infrastructure and resources facilitated the integration of advanced technology into therapy. Evidence points to supportive organisational systems and appropriate skill-mix where advanced technologies are embedded in routine workflows.^31–39,44,46–48,50,51^ Studies also describe the physical environment arranged to support engagement (quiet, well-equipped rooms) to optimise the sensory experience and support therapeutic engagement.^40^ While adoption of robotics/VR suggests potential for innovation/risk-taking, the reports provide little detail on effective staff relationships, power sharing, or organisational systems for shared decision-making.

### 3.4. Person-centred processes

Engaging authentically and shared decision-making is evidenced through personalised parameter setting, speed, step length, body-weight support, according to individual preferences, tolerance, comfort, and progress.^31–38,41,42,44–47,49–51^ Working with beliefs and values is perceived where preferred music or visual/avatar feedback is used to co-design training moments.^33,39,40,48^ Designs that align stimulation or training to functional goals demonstrate alignment with working holistically.^43,50,51^ Direct evidence of sympathetic presence is suggested but not explicitly measured.

### 3.5. Outcomes

All included studies reported outcomes. Across studies, outcomes were predominantly assessed using performance-based functional measures, capturing motor function,^31–51^ cognitive function^32^ and capacity to perform activities of daily living.^38^ One study additionally reported a biomarker outcome (serum brain-derived neurotrophic factor),^42^ and two studies included PROMs focused on quality of life.^32,40^ No PREMs were reported in any of the included studies. Evidence related to the development of a healthful culture was not described within the study reports.

## 4. Discussion

This analysis offers a novel perspective on integrating person-centred care principles into technology-based gait rehabilitation for stroke survivors. By examining randomised controlled trials through the lens of the PCPF, we identified that several domains of the framework were represented, most notably *Person-Centred Processes*, followed by *Prerequisites* and the *Practice Environment*. However, this representation was uneven: at the construct level, only selected elements within each domain were identified, rather than the full set of constructs that collectively define person-centred practice within the framework.

The complete absence of the *Macro Context* domain across all studies raises important questions about the broader systemic readiness to embed person-centredness into technologically driven rehabilitation models. The *Macro Context* encompasses policy frameworks, strategic leadership, workforce development, and strategic frameworks, shaping the sociopolitical conditions in which PCP can thrive.^52^ In principle, the justification for developing or testing such technology-based interventions could derive directly from macro-level drivers such as national rehabilitation strategies or funding priorities. However, none of the included trials referenced these structural enablers. Without alignment to broader policy and system-level frameworks, even clinically effective interventions may face challenges related to scalability, equitable access, and long-term sustainability, particularly in contexts of shifting organisational or funding priorities.^53,54^

At the same time, these findings must be interpreted with appropriate caution. The apparent absence of the *Macro Context* domain may reflect, in part, the methodological focus and reporting conventions of randomised controlled trials rather than the full complexity of practice realities. It may also indicate a level-of-analysis misalignment between macro-level constructs within the PCPF and the meso- and micro-level focus typical of intervention trials. Randomised controlled trials are primarily designed to demonstrate intervention efficacy under controlled conditions and therefore tend to privilege internal validity, standardisation, and measurable clinical outcomes over contextual, organisational, or relational dimensions of care.^55,56^ Elements related to the *Macro Context* may be present in practice but remain underreported or implicitly assumed within trial publications. Accordingly, the findings of this review should be interpreted as reflecting how person-centredness is articulated and operationalised within the published randomised controlled trial literature, rather than as a comprehensive representation of person-centred activity in real-world technology-based gait rehabilitation. Explicitly acknowledging this distinction strengthens interpretive balance while underscoring the need for more comprehensive reporting and theory-informed study designs.

The most represented domain was *Person-Centred Processes*, but closer examination reveals important nuances. Several interventions included elements that appear to tailor training parameters to individual capabilities and progress, or to provide real-time feedback to encourage active engagement and self-correction.^31,33,34,37–40,45–49^ However, in many cases, this “personalisation” was primarily a functional necessity, adjusting speed, load, or stimulation parameters to match physical tolerance, rather than an intentional enactment of person-centred principles such as authentic engagement or shared decision-making.^22^ Notably, none of the studies explicitly reported eliciting participant input into goal setting, training preferences, or motivational strategies. This distinction is critical: if adjustments are framed purely as clinical pragmatism or device optimisation, they risk being perceived and implemented as secondary or incidental, thereby limiting the systematic integration of person-centred approaches into program design, implementation, and evaluation. From the PCPF’s perspective, authentic connection, opportunities for patient involvement, shared decision-making, and trust-building may be eroded, reducing the sense of partnership between professionals and participants. Furthermore, when individual values, goals, and lived contexts are not explicitly integrated, interventions may feel impersonal or mechanistic.

In the *Prerequisites* domain, there was evidence of professional competence: clinicians demonstrated the technical expertise required to calibrate robotic systems,^31–33,35–39^ adapt electrical and auditory stimulation parameters,^40–46^ and monitor treadmill workloads in real time.^34,47^ These skills align with the professionally competent attribute of the PCPF, insofar as they require integrating advanced technical knowledge with sound clinical judgment, vigilant attention to safety, and real-time accountability. However, other attributes, such as developed interpersonal skills, commitment to the job, clarity of beliefs and values, and knowing self, were not evident in the descriptions of any intervention. This absence reflects a broader limitation in technology-focused rehabilitation research, where relational and reflective capacities of practitioners are rarely acknowledged, measured, or theorised despite their known importance to PCP.

*The Practice Environment* domain was less frequently represented and, where present, was primarily confined to features of the physical environment (e.g., well-equipped facilities, access to advanced devices).^31–33,35–39^ There was no evidence of appropriate skill mix*,* shared decision-making systems, effective staff relationships, supportive organisational systems, or power-sharing arrangements.^22^ The under-representation of broader environmental attributes may reflect the constraints of randomised controlled trials reporting, which typically prioritise methodological and intervention details over contextual or organisational factors. Omitting discussion of organisational culture, leadership support, team effectiveness, and resource allocation represents a missed opportunity to understand the feasibility and sustainability of embedding person-centred principles in technological rehabilitation.

The *Outcomes* domain was consistently represented, although predominantly in functional terms. All interventions reported the intention to assess improvements in gait speed, balance, and mobility, which are standard indicators of functional recovery. Notably, no PREMs were identified in any of the included trials. These findings suggest that “success” in technology-based gait rehabilitation continues to be primarily defined through a biomedical and performance-oriented lens. From a PCPF perspective, person-centred outcomes extend beyond functional gains to include the cultivation of a healthful culture that enables human flourishing.^22^ Evaluations should therefore pair performance metrics with both outcome- and experience-focused measures, so that success reflects what matters to the person as well as what improves their function. The absence of PREMs may also reflect practical and methodological challenges associated with embedding experience-focused measures within highly protocolised, device-driven trials, including respondent burden, timing of data collection during intensive interventions, and the traditional prioritisation of performance endpoints in rehabilitation technology research.

The observation that, across the different PCPF domains, the data identified in the included studies addressed only selected constructs suggests that evidence of person-centredness was confined mainly to isolated domain components rather than a comprehensive enactment of all constituent constructs. In most cases, although a domain could be identified, many of its defining constructs were neither described nor operationalised. This pattern indicates that the integration of person-centred principles in the included studies was more incidental than intentional. This limits the capacity to monitor and optimise interventions against established person-centred benchmarks, leaving gaps in evidence about their relational, ethical, and experiential impact. As well, it risks perpetuating a narrow definition of success based on biomechanical or functional gains, while disconnecting from the individual realities, priorities, and goals of those receiving care.

### 4.1. Implications

The findings of this review highlight a critical gap in the integration of person-centred care principles in technology-based gait rehabilitation for stroke survivors. Herein, we propose implications for clinical practice and future research.

#### 4.1.1. Clinical practice implications

Training parameters should be personalised and actively incorporate individual goals, preferences, and values into the delivery of interventions. This requires recognising the person as an active partner rather than a passive recipient of care, a key aspect of our applied framework. Furthermore, skilled practitioners are needed for safe and effective use of complex devices. Clinical competence must be complemented by interpersonal skills, reflective practice, and a clear articulation of values, which are rarely documented in randomised controlled trials but are central to sustained professional accountability, patient engagement, and trust-building with the person undergoing rehabilitation. Sustainable integration of person-centred care requires alignment between individual competencies, organisational culture, and systemic conditions. Without supportive leadership, investment in resources, and workforce development, even well-designed interventions risk being unsustainable when scaled up or transferred to less resourced contexts. Clinical teams therefore have a dual responsibility: to deliver person-centred processes in direct care and advocate for organisational and policy structures that enable these processes to prevent technological innovations from overshadowing the relational dimensions of care. Lastly, the need to integrate PROMs and PREMs will ensure that intervention success reflects functional recovery and the person’s experience and well-being.

#### 4.1.2. Future research implications

There is a clear need for rehabilitation research and practice to adopt theoretical frameworks such as the PCPF as prospective design frameworks, rather than retrospective analytic tools.^57^ Doing so would allow for deliberate integration of person-centred domains into technology-based interventions from the outset, ensuring that technological innovation serves to enhance the relational and humanistic dimensions of care. While technology-based gait rehabilitation for stroke survivors demonstrates significant potential to embody elements of PCP, this potential is far from fully realised. Person-centredness in these interventions should be intentional, explicit, and systematically embedded across all domains of the PCPF, from care to macro-level policy alignment.

Formal person-centred intervention protocols within technology-based rehabilitation programmes should be established. This will ensure all stages embed PCP domains – from intervention design to assessment and evaluation. Future trial reporting should capture contextual, organisational, and relational factors, enhancing reproducibility and scalability. Researchers should consistently prioritise broader person-centred outcomes, incorporating both PROMs and PREMs. We also encourage the uptake of mixed methods in intervention design. Mixed-methods designs can combine efficacy metrics with qualitative insights to enhance understanding of the relational and experiential dimensions of PCP. Lastly, researchers should study the applicability of interventions across diverse cultural and health system contexts, including low-income and middle-income economies, to ensure global relevance.

### 4.2. Strengths and limitations

Our approach, applying the PCPF to a corpus of randomised controlled trials, offers a novel lens for interpreting existing evidence, moving beyond conventional efficacy metrics to consider the relational, contextual, and experiential dimensions of care. The structured examination of trial descriptions enabled the identification of implicit person-centred elements that might otherwise remain under-recognised in the literature, yielding insights that are conceptually grounded and practically relevant for the design of future interventions.

However, several limitations should be acknowledged. First, the analysis relied exclusively on the information reported in published trial descriptions, which may have omitted relevant details about person-centred approaches. It is possible that certain PCPF domains were present in the interventions but not explicitly documented. Additionally, the retrospective nature of the analysis means that the PCPF was applied post hoc to studies not originally designed to evaluate person-centredness, and the identification of domains was therefore based on reported intervention characteristics rather than on prospectively measured person-centred outcomes. Moreover, trial reports rarely provided sufficient detail to determine whether device parameter adjustments reflected standardised, protocol-driven optimisation or intentional, person-centred processes, further limiting conclusions regarding the enactment of person-centredcare.

Second, the decision to include only randomised controlled trials may have influenced the pattern of findings. While this design provides rigorous evidence regarding intervention effects and detailed protocol descriptions, it may be less sensitive to relational, contextual, and experiential dimensions that are more commonly explored in qualitative, mixed-methods, feasibility, or implementation research. Consequently, the limited explicit reporting of person-centred elements should be interpreted with caution, as it may partly reflect the epistemological orientation and reporting conventions of RCTs rather than the actual absence of person-centred practices in technology-based gait rehabilitation.

Finally, this review deliberately focused on efficacy-oriented trials to examine how person-centredness is represented in high-level intervention evidence. Future work would benefit from complementary syntheses including qualitative and implementation-focused studies to provide a more comprehensive understanding of how person-centred principles are operationalised and experienced in technology-based gait rehabilitation.

## 5. Conclusion

This analysis reveals that technology-based gait rehabilitation after stroke incorporates aspects of PCP. However, these are applied inconsistently across the domains of the PCPF. The strongest representation was in *Person-Centred Processes*, with limited attention to *Prerequisites* and *the Practice Environment*, and no evidence of *Macro Context* considerations. Personalisation often appeared to be a by-product of clinical pragmatism or device functionality, rather than a deliberate commitment to person-centred principles, and broader outcomes, such as autonomy, participation, and quality of life, were seldom prioritised.

Progress in this field will require more intentional trial design. Future randomised controlled trials should prospectively embed the PCPF by explicitly specifying how person-centred processes are operationalised within intervention protocols, incorporating routine measurement of both functional and experience-focused outcomes, and documenting how individual goals and preferences inform intervention tailoring and progression. Greater attention to the organisational and policy context in trial reporting, such as workforce preparation, implementation settings, and service integration pathways, will also be necessary to support scalability and sustainability. Without these shifts in trial design and reporting, evidence from technology-based gait rehabilitation risks advancing functional performance while underrepresenting the relational and experiential dimensions of care.

## Supplemental material

Supplemental material - Exploring person-centredness in technology-based gait rehabilitation after stroke: A scoping review framework analysis Supplemental material for Exploring person-centredness in technology-based gait rehabilitation after stroke: A scoping review framework analysis by Júlio Belo Fernandes, Diana Vareta, Sónia Fernandes, Ana Chalaça, Ana Silva Almeida, Ana Catarina Maia, Faiza Magsi, Steven Hall and Brendan McCormack in DIGITAL HEALTH.

## Data Availability

The data that support the findings of this study are available from the corresponding author upon reasonable request.*
