# Supplementary material for: Exploring person-centredness in technology-based gait rehabilitation after stroke: A scoping review framework analysis
Source: Digit Health. 2026 Apr 30;12:20552076261443750. doi: 10.1177/20552076261443750 (PMC13153535; doi:10.1177/20552076261443750)
Supplement: Supplemental material - Exploring person-centredness in technology-based gait rehabilitation after stroke: A scoping review framework analysis [file sj-pdf-1-dhj-10.1177_20552076261443750.pdf]

Table 1: Study characteristics

Authors / Year/ Title	Aim	PCCF
Cha et al. (2014)<br>Intensive gait training with rhythmic auditory stimulation in individuals with chronic hemiparetic stroke: a pilot randomized controlled study.	To investigate the effect of intensive gait training with rhythmic auditory stimulation on postural control and gait performance in individuals with chronic hemiparetic stroke.	**Prerequisites**<br>• technical competence to adjust cadence and rhythm.<br>**Practice Environment**<br>• a quiet and well-equipped room to enhance sensory experience.<br>**Person-Centred Processes**<br>• patient involvement in care, through choice of preferred music and adaptation of training to individual rhythm.<br>**Outcomes**<br>• performance-based outcomes (motor performance).<br>• PROMs (quality of life).
Drużbicki et al. (2015)<br>Efficacy of gait training using a treadmill with and without visual biofeedback in patients after stroke: A randomized study.	To evaluate the effects of gait training using a treadmill with and without visual biofeedback in patients in the late period after stroke, and to compare both training methods.	**Prerequisites**<br>• individual assessment and adaptation by the professional.<br>**Person-Centred Processes**<br>• progressive adjustment of speed and step length according to tolerance, comfort, and capacity<br>**Outcomes**

		• performance-based outcomes (motor performance).
Hwang et al. (2015)<br>Treadmill training with tilt sensor functional electrical stimulation for improving balance, gait, and muscle architecture of tibialis anterior of survivors with chronic stroke: A randomized controlled trial.	To investigate the effects of the treadmill training with tilt sensor functional electrical stimulation on the balance, gait, and muscle architecture of the tibialis anterior in stroke survivors.	**Prerequisites**<br>• continuous assessment to optimise training.<br>**Person-Centred Processes**<br>• individual adjustment of electrical stimulation intensity and treadmill speed according to comfort, performance, and tolerance.<br>**Outcomes**<br>• performance-based outcomes (motor performance).
Bang & Shin (2016)<br>Effects of robot-assisted gait training on spatiotemporal gait parameters and balance in patients with chronic stroke: A randomized controlled pilot trial.	To compare the effects of robot-assisted gait training versus treadmill gait training on spatiotemporal gait parameters, balance, and activities-specific balance confidence in stroke patients.	**Prerequisites**<br>• competence to calibrate weight support and intensity, and the ability to ensure the person's safety and convey confidence.<br>**Practice Environment**<br>• specialised robotic platform integrated.<br>**Person-Centred Processes**<br>• continuous personal feedback and encouragement to synchronise movements with the device.<br>**Outcomes**<br>• performance-based outcomes (motor performance).

Choi et al. (2017)<br>Whole-Body Vibration Combined with Treadmill Training Improves Walking Performance in Post-Stroke Patients: A Randomized Controlled Trial.	To investigate the effect of whole-body vibration combined with treadmill training on walking performance in patients with chronic stroke.	**Practice Environment**<br>• specialised platform<br>**Person-Centred Processes**<br>• gradual progression and focus on functional capacity.<br>**Outcomes**<br>• performance-based outcomes (motor performance).
Dujović et al. (2017)<br>Novel multi-pad functional electrical stimulation in stroke patients: A single-blind randomized study.	To evaluate efficacy of additional novel functional electrical stimulation system to conventional therapy in facilitating motor recovery in the lower extremities and improving walking ability after stroke.	**Prerequisites**<br>• calibration and adaptation of parameters.<br>**Person-Centred Processes**<br>• stimulation synchronised with the gait cycle.<br>**Outcomes**<br>• performance-based outcomes (motor performance).
Moon & Kim (2017)<br>Effect of three-dimensional spine stabilization exercise on trunk muscle strength and gait ability in chronic stroke patients: A randomized controlled trial.	To investigate the effects of the newly developed Spine Balance three-dimensional system on trunk strength and gait abilities of chronic stroke patients.	**Prerequisites**<br>• calibration and adaptation of parameters.<br>**Practice environment**<br>• three-dimensional spine-stabilizing system.<br>**Person-Centred Processes**<br>• adaptation of training to functional and balance needs.<br>**Outcomes**<br>• performance-based outcomes (motor performance).

Sharif et al. (2017)<br>Effectiveness of Functional Electrical Stimulation versus Conventional Electrical Stimulation in Gait Rehabilitation of Patients with Stroke.	To compare the effectiveness of functional electrical stimulation versus conventional electrical<br>stimulation in gait rehabilitation of patients with stroke for finding the most appropriate problem-oriented treatment for foot drop patients.	**Person-Centred Processes**<br>• stimulation synchronised with gait phase, relevant to the person's function and comfort.<br>**Outcomes**<br>• performance-based outcomes (motor performance).
Park & Chung (2018)<br>The effects of robot-assisted gait training using virtual reality and auditory stimulation on balance and gait abilities in persons with stroke.	To investigate the effects of robot-assisted gait training using virtual reality and<br>auditory stimulation on balance and gait abilities in stroke patients.	**Prerequisites**<br>• calibration and adaptation of parameters.<br>**Practice Environment**<br>• specialised robotic platform integrated.<br>**Person-Centred Processes**<br>• use of virtual reality and auditory cues to enhance motivation and engagement.<br>**Outcomes**<br>• performance-based outcomes (motor performance).
Mustafaoglu et al. (2020)<br>Does robot-assisted gait training improve mobility, activities of daily living and quality of life in	To investigate the effects of robot-assisted gait training on mobility, activities of daily living, and quality of life in stroke rehabilitation.	**Prerequisites**<br>• calibration and adaptation of parameters.<br>**Practice Environment**<br>• specialised robotic platform integrated.

stroke? A single-blinded, randomized controlled trial.		**Person-Centred Processes**<br>• progressive adaptation of speed and support according to tolerance.<br>**Outcomes**<br>• performance-based outcomes (motor performance).
Kang et al. (2021)<br>Walking Training with a Weight Support Feedback Cane Improves Lower Limb Muscle Activity and Gait Ability in Patients with Chronic Stroke: A Randomized Controlled Trial.	To investigate the effect of walking training with a weight support feedback cane on lower limb muscle activity and gait ability of chronic stroke patients.	**Prerequisites**<br>• calibration and adaptation of parameters.<br>**Person-Centred Processes**<br>• real-time feedback to encourage weight transfer to the affected limb.<br>**Outcomes**<br>• performance-based outcomes (motor performance).
Kooncumchoo et al. (2021)<br>Gait Improvement in Chronic Stroke Survivors by Using an Innovative Gait Training Machine: A Randomized Controlled Trial.	To evaluate the effects of a newly invented gait training machine (I-Walk) on lower limb function and gait performance in chronic stroke individuals.	**Prerequisites**<br>• professional competence to monitor and adapt training.<br>**Practice Environment**<br>• specialised gait training platform.<br>**Person-Centred Processes**<br>• support and cadence adjustment and progression based on tolerance.<br>**Outcomes**

		• performance-based outcomes (motor performance).
Cho et al. (2022)<br>Does electrical stimulation synchronized with ankle movements better improve ankle proprioception and gait kinematics in chronic stroke? A randomized controlled study.	To investigate the effects of passive biaxial ankle movement training synchronized with electrical stimulation therapy on ankle proprioception, passive range of motion, and strength, balance, and gait of chronic stroke patients.	**Prerequisites**<br>• technical skill to synchronise electrical stimulation with specific joint movement.<br>**Person-Centred Processes**<br>• stimulation according to participants' tolerance level<br>**Outcomes**<br>• performance-based outcomes (motor performance).
Choi (2022)<br>Effects of Robot-Assisted Gait Training with Body Weight Support on Gait and Balance in Stroke Patients.	To investigate the effects of robot-assisted gait training with body weight support on gait and balance in stroke patients.	**Prerequisites**<br>• professional competence to monitor and adapt training.<br>**Practice Environment**<br>• specialised robotic platform integrated.<br>**Person-Centred Processes**<br>• individual adjustment of treadmill speed and robotic guidance.<br>**Outcomes**<br>• performance-based outcomes (motor performance).
Shin & Chung (2022)<br>The effects of treadmill training with visual feedback and rhythmic	To investigate the effect of treadmill training with visual feedback and rhythmic auditory cue for walking	**Prerequisites**<br>• professional competence to monitor and adapt training.<br>**Practice Environment**

auditory cue on gait and balance in chronic stroke patients: a randomized controlled trial.	symmetry on spatiotemporal gait parameters and balance abilities.	• integration of advanced feedback systems into rehabilitation.<br>**Person-Centred Processes**<br>• real-time feedback promoting self-correction and active engagement.<br>**Outcomes**<br>• performance-based outcomes (motor performance).
Castelli et al. (2023)<br>Robotic-assisted rehabilitation for balance in stroke patients (ROAR-S): effects of cognitive, motor and functional outcomes.	To evaluate the effects of rehabilitation with Hunova on cognitive function and balance in older adults with stroke.	**Prerequisites**<br>• professional competence to monitor and adapt training.<br>**Practice Environment**<br>• specialised robotic platform integrated into conventional therapy.<br>**Person-Centred Processes**<br>• parameters were adjusted according to each participant.<br>**Outcomes**<br>• performance-based outcomes (motor and cognitive performance).<br>• PROMs (quality of life).
da Cunha et al. (2024)	To investigate the effects of combining transcranial direct current stimulation	**Prerequisites**<br>• professional competence to monitor and adapt training.

tDCS does not add effect to foot drop stimulator and gait training in improving clinical parameters and neuroplasticity biomarkers in chronic post-stroke: randomized controlled trial.	and foot drop stimulators on motor impairment, functional mobility, and brain-derived neurotrophic factor serum levels.	**Person-Centred Processes**<br>• parameters were adjusted according to each participant.<br>**Outcomes**<br>• performance-based outcomes (motor performance).<br>• biomarker outcomes (blood measures).
Wang et al. (2024)<br>Repetitive Transcranial Magnetic Stimulation Coupled With Visual-Feedback Cycling Exercise Improves Walking Ability and Walking Stability After Stroke: A Randomized Pilot Study.	To determine the effects of repetitive transcranial magnetic stimulation coupled with cycling exercise on walking ability and stability in patients with stroke and explore the potential mechanisms underlying motor cortex recovery.	**Prerequisites**<br>• professional competence to monitor and adapt training.<br>**Practice Environment**<br>• integration of advanced feedback systems into rehabilitation.<br>**Person-Centred Processes**<br>• parameters were adjusted according to each participant.<br>**Outcomes**<br>• performance-based outcomes (motor performance).
Jang et al. (2025)<br>Effect of active over-ground body weight-support walking system on balance and gait ability in stroke: A randomized controlled trial	To examine the effects of gait training using an active over-ground body weight-support walking system on balance and gait in stroke patients.	**Prerequisites**<br>• professional competence to monitor and adapt training.<br>**Practice Environment**<br>• specialised robotic platform integrated<br>**Person-Centred Processes**

		• parameters were adjusted according to each participant.<br>**Outcomes**<br>• performance-based outcomes (motor performance).
Fang et al. (2025)<br>Effects of combining rTMs and augmented reality gait adaptive training on walking function of patients with stroke based on three-dimensional gait analysis and sEMG: a randomized controlled trial.	To investigate the effects of combining repetitive transcranial magnetic stimulation and augmented reality gait adaptive training on motor function in survivors of stroke.	**Prerequisites**<br>• professional competence to monitor and adapt training.<br>**Practice Environment**<br>• intelligent gait training system<br>**Person-Centred Processes**<br>• parameters were adjusted according to each participant.<br>**Outcomes**<br>• performance-based outcomes (motor performance).
Park (2025)<br>Comparison of the effects of robot-assisted gait training using FES versus virtual reality for stroke patients. A randomized clinical trial.	To compare the effects of robot-assisted gait training using Functional electrical stimulation and Virtual Reality on balance and gait abilities in stroke patients and to suggest an effective treatment strategies.	**Prerequisites**<br>• professional competence to monitor and adapt training.<br>**Practice Environment**<br>• specialised robotic platform integrated.<br>**Person-Centred Processes**<br>• parameters were adjusted according to each participant.<br>**Outcomes**<br>• performance-based outcomes (motor performance and capacity to perform activities of daily living).
